# Productivity-adjusted life-years and correlates of uncontrolled hypertension at two health facilities in Zambia

**DOI:** 10.1371/journal.pone.0295401

**Published:** 2023-12-07

**Authors:** Joreen P. Povia, Sepiso K. Masenga, Benson M. Hamooya, Yordanos Gebremeskel

**Affiliations:** 1 Department of Economics, School of Social Sciences, Mulungushi University, Kabwe, Zambia; 2 HAND Research Group, School of Medicine and Health Sciences, Mulungushi University, Livingstone Campus, Livingstone, Zambia; AIIMS Jodhpur: All India Institute of Medical Sciences - Jodhpur, INDIA

## Abstract

**Background:**

Hypertension has in the recent past surfaced as one of the conditions that has a significant impact on workforce productivity in emerging economies. Zambia is no different and has in the recent past recorded increasing cases. Despite the impact of hypertension being of great importance in regards to productivity, we have scarcity of data and studies on hypertension-related Productivity-Adjusted Life-Years (PALYs) in Zambia and Africa at large.

This study assessed the impact of hypertension on PALYs lost and socioeconomic factors associated with nonadherence to antihypertensive medication (NATAM).

**Methods:**

This was a cross-sectional study of 198 participants from Livingstone University Teaching Hospital and Maramba Clinic situated in Livingstone, Zambia. Structured questionnaires were used to collect data. Productivity index multiplied by years lived was used to calculate PALYs and descriptive statistics were used to summarize sociodemographic, clinical and economic variables. Multivariable logistic regression was used to determine factors associated with NATAM.

**Results:**

The participants had a median age (interquartile range (IQR)) of 49 years (41, 59) and 60.1% (n = 119) were females while 39.9% (n = 79) were male. Our estimated PALYs lost per person due to hypertension were 0.2 (IQR 0.0, 2.7). Cumulative PALYs value lost due to the burden of hypertension was estimated to be at $871,239.58 in gross domestic product (GDP). The prevalence of NATAM was 48% (n = 95). The factors that were significantly associated with NATAM were age (odds ratio (OR) 0.94; 95% confidence interval (CI) 0.90, 0.98), female sex (OR 2.52; 95%CI 1.18, 5.40), self-employment (OR 2.57; 95%CI 1.02, 6.45) and absenteeism from work (OR 3.60; 95%CI 1.16, 11.22).

**Conclusions:**

Findings in our study highlight a high economic loss of PALYs due to hypertension with a potential to impact GDP negatively. We also found that NATAM reduced productivity and income among individuals of working age further impacting PALYs lost due to hypertension. The factors associated with NATAM were age, sex, employment status and absenteeism from work. This study underscores the need for interventions targeting young people, females, self-employed individuals, and absentees at work to improve adherence to antihypertensive drugs in order to reduce PALYs lost due to hypertension.

## Background

In the recent past, it has been noted that health has a big impact on employment as it affects productivity [1,2]. Employees with ill health conditions are more likely to cause losses to a company’s production owing to days absent from work compared to healthy individuals [3,4]. Due to uncontrolled hypertension resulting from nonadherence to antihypertensive medication (NATAM), companies make losses in production as employees face disabilities and premature death [2,5–7].

The global prevalence of hypertension among adults is higher in low and middle-income countries (LMICs) (31.5%, 1.04 billion people) when compared to high-income countries (28.5%, 349 million people) as of 2010 [8]. This is due in part, to the lack of hypertension awareness, treatment and control of blood pressure (BP) [8]. In sub-Saharan Africa (SSA), the burden of hypertension was disproportionately higher with a significant number undiagnosed [7]. Hypertension or high BP is a risk factor for stroke, heart attack and death [9,10]. The risk posed by hypertension is attenuated by use of antihypertensive medication to control BP [11]. Hence, persons living with hypertension are required to take their medication daily to avoid adverse cardiovascular events. However, the cost of medication and socioeconomic status (SES) has been linked to nonadherence to antihypertensive medication (NATAM) [12,13]. Other factors contributing to NATAM include old age, marital status, stress, obesity, being widowed/separated, smoking, having normal BP readings, lack of social support [14–17]. Further, low SES is associated with higher disease costs which lowers the quality of life as huge amounts of money are instead channeled to medical bills [18,19].

Uncontrolled hypertension resulting from NATAM has the capability of reducing productivity in accumulated work days lost owing to ill health that results in absenteeism and reduced efficiency at work (presenteeism) and therefore, increasing productivity-adjusted life-years (PALYs) lost due to hypertension [5]. The resulting loss of productivity can potentially impose an economic burden on individuals, employees and governments through reduced earnings, tax revenues, and gross domestic product (GDP) [20]. The economic burden of hypertension is one that is real and continues to affect economies world over [8,21]. In the United States, the estimated direct and indirect costs due to hypertension amounted to $37.2 billion -$50.3 billion in direct medical costs and were estimated to be at $13.1 billion in indirect costs caused by the loss of productivity due to hypertension [22]. Costs related to treating cardiovascular disease caused by hypertension were estimated to be between $351.8 billion to 209.3 billion in direct costs and $142.5 billion in indirect costs due to lost productivity [22]. In Sub-Saharan Africa, medication costs associated with hypertension ranged from 1.70$ to 97.06$ per month from a patient perspective whereas from provider perspective, the cost could be as high as 193.55$ per patient per month [23]. In another study from southern Ethiopia, they found that the economic burden of hypertension was $105.55 per person per month, an equivalent of $514 232.16 total [24]. A study by Kaiser et al., from Zambia found that the cost of antihypertensive medication in local pharmacies was even higher than their international reference prices [25]. Thus, high cost of medication could be an obstacle to medication adherence in persons with hypertension, a factor that can increase NATAM resulting in adverse outcomes [26]. These data are evident that hypertension is becoming one of the diseases of public health concern and if not curbed will deprive economies of sustained and efficient work force.

Despite the fact that the modifiable risk factors and interventions are known, hypertension is still considered one of the long-standing challenging noncommunicable diseases (NCDs) in Zambia [27]. In Zambia, NCDs accounted for 23% of the deaths in 2008 [28] and 29% of all deaths by 2016 [29]. It is also expected that there will be a rapid growth of NCDs in the near future [7,28]. Although hypertension is attenuated by the use of anti-hypertensive medication, a large proportion of hypertensive patients are not adherent to anti-hypertensive medication [30]. At the University Teaching Hospital (UTH) in Zambia, the prevalence of NATAM was 30% [30]. Findings revealed that patients who were less likely to adhere to antihypertensive medication were those taking one drug, living more than 10km from the hospital and those who did not find prescribed medication at the hospital pharmacy thereby requiring purchase. However, there is limited information regarding the magnitude and correlates of NATAM, and the impact of hypertension on PALYs in Zambia. Understanding and mitigating the predictors and burden of NATAM in Zambia has potential to improve PALYs by reducing absenteeism and presenteeism associated with morbidity, improve productivity, reduce lost GDP due to the burden of disease and further reduce the costs incurred due to terminal diseases caused by hypertension. We, therefore, investigated the prevalence of NATAM, its correlates and also determined the PALYs lost due to hypertension.

## Methods

### Study design and population

This was a cross-sectional study conducted at Livingstone University Teaching Hospital (LUTH) and Maramba clinic among adults with hypertension between April 2023 and June 2023. LUTH and Maramba health facilities are all located in the vicinity of the Livingstone city town area with LUTH being situated closer to the town than Maramba clinic.

### Eligibility and recruitment

We purposively selected male and female adult participants who were 18 years and above who were attending routine hypertension clinics at LUTH and Maramba clinic and were living with hypertension. The two health facilities were selected due to the high volume of persons living with hypertension enrolled in care and having an existing and well organized hypertension clinic. The two facilities are near to each other and serve communities with almost similar characteristics. During routine clinic visits, the attending clinician explained and provided the necessary information to the participants prior to recruitment. All participants had to sign consent forms before they were included in the study. Persons with disabilities and pregnant women are more likely to be absent from work or even suffer from presenteeism due to their condition which may not necessarily be associated with hypertension. We therefore excluded them from the study in order to eliminate the confounding effect associated with their condition. We also excluded participants who did not provide information on the outcome.

### Sample size calculation

We used www.OpenEpi, a free online software to estimate sample size using the formulae below:

$$Sample\;size\;N=\frac{\left[\left(DEFF*Np(1-p\right)\right]}{\left[\left(\frac{d^{2}}{\;Z_{1-\frac{\alpha }{2}}^{2}}*(N-1)+p*(1-p\right)\right]}$$

Where N = Population size (for finite population correction factor or fpc)(N): 450

Hypothesized % frequency of outcome factor in the population (p) from a study conducted in Zambia [30]: 30%+/-5

Confidence limits as % of 100(absolute +/- %) (d): 5%

Design effect (for cluster surveys-DEFF): 1

The minimum total sample size required was 189 at 95% confidence level. We enrolled 198 participants.

### Ethics approval and consent to participate

Ethical approval was obtained from the Mulungushi University School of Medicine and Health Sciences Research Ethics Committee (IRB: 00012281 FWA: 0002888) on 5^th^ April 2023 and from the Zambia National Health Research Authority on 17^th^ April 2023. Participants provided informed consent which was obtained as signed written consents before they were recruited into the study. Recruitment of participants from the study centers started on 18^th^ April 2023 until 1^st^ June 2023.

### Data collection

Sociodemographic and clinical data were collected using a structured questionnaire with the help of medical personnel. Additional information was abstracted from patient files. Study data were collected and managed using Research Electronic Data Capture (REDCap) tools [31,32]. REDCap is a secure, web-based software platform designed to support data capture for research studies, providing 1) an intuitive interface for validated data capture; 2) audit trails for tracking data manipulation and export procedures; 3) automated export procedures for seamless data downloads to common statistical packages; and 4) procedures for data integration and interoperability with external sources [31,32].

### Study variables

Nonadherence to antihypertensive medication (NATAM) is the response (outcome) variable. We used the hill-bone scale [33] categorized into participants who are completely adherent and those nonadherent to antihypertensive medication. Diagnosis of hypertension was based on systolic and diastolic blood pressure ≥ 140/90 mmHg on more than 2 occasions or a history of antihypertensive medication usage [34]. Independent variables included demographic, social, economic and clinical characteristics. Absenteeism was defined as having been absent from work due to hypertension related illness for at least one day whereas presenteeism was defined as reduced efficiency reported by being present at the work place but fail to work on at least one day.

### Determination of PALYs

To calculate PALYs, we multiplied productivity index by the years lived with a range from 0 to 1 where:0 (entirely unproductive) and 1(entirely productive without absenteeism or presenteeism). The economic cost of one productivity-adjusted life-year was equated to the GDP per capita of Zambia as of 2021 amounting to $1,137.34 as recorded by *macrotrends*.*net*. This estimation was adapted from a study by *Hird et al*., [5].

The current total number of working days in Zambia is 245 days.


$$productivity\;index=\frac{number\;of\;working\;days\;per\;year-number\;of\;days\;absent\;per\;year}{Number\;of\;working\;days\;per\;year}$$

### Data analysis

We used the productivity index multiplied by participant age to assess the impact of hypertension on PALYs among participants of working age. To enable us treat each participant with hypertension as their own control (without hypertension) for purposes of estimating the number of PALYs “lost” due to hypertension we computed the difference between the PALYs when the participants are assumed to be healthy (without hypertension) and the PALYs when the same individuals are living with hypertension. This difference between the assumed PALYs and the estimated PALYs is significant as it highlights the aggregate loss to production suffered due to disease and therefore provides a basis for policy makers and health providers to realize the effects of the burden of disease on production [35,36].

Descriptive statistics (medians, frequencies, proportions, and interquartile ranges) were used to describe the distribution of variables of interest among the study participants. As data were approximately not normally distributed, we used Wilcoxon rank sum test (to compare medians between two groups; the test of normality used was Shapiro-Wilk test). The chi-square test was used to ascertain a relationship between the outcome variable and categorical independent variables. Logistic regression model (bivariate and multivariable) was used to examine factors associated with NATAM. All variables in this study were selected based on economic expertise and previous literature. We included in the final model, all variables that were significant on univariable analysis including age and sex. The continuous variable “days with presenteeism at work” was not included in the final model despite being significant at univariable analysis because the dichotomized one “presenteeism”, is more preferred and was also significant on univariable analysis. Hence only presenteeism was added to avoid duplication of effects. A p-value of less than 0.05 was considered to be statistically significant. Statistical Package for the Social Sciences (SPSS) was used for data analysis.

### Reporting format

We have used the strengthening the reporting of observational studies in epidemiology (STROBE). See S1 Table for details.

## Results

### Basic characteristics of study participants

A total of 198 participants were recruited with a median age (interquartile range; min-max) of 49 (41.0–59.0; 23–82) years, with a female preponderance of 60.1% (n = 119), Table 1. The majority were from Livingstone University Teaching Hospital (n = 136, 68.7%), were married (n = 137, 69.2%), in self-employment (n = 39, 19.7%), and had obtained a tertiary education (n = 77, 39.1%).

**Table 1:** Demographic and socioeconomic characteristics of study participants.

Characteristics	Frequency or Median	Percentage or Interquartile range
**Age,** *years*	49	(41, 59)
**Sex**		
*Male*	79	39.9
*Female*	119	60.1
**Facility**		
LUTH	136	68.7
Maramba	62	31.3
**Marital status, n = 196**		
*Married*	137	69.2
*Single*	20	10.1
*Widowed*	21	10.6
*Divorced*	15	7.6
*Separated*	3	1.5
**Employment Status**		
*GRZ employee*	39	19.7
*Non-GRZ Employee*	22	11.1
*Self employed*	74	37.4
*Student*	3	1.5
*Retired*	15	7.6
*Unemployed (able to work)*	28	14.1
*Unemployed (Unable to work)*	17	8.6
**Education Status, n = 197**		
*No formal schooling*	7	3.6
*Primary*	54	27.4
*Secondary*	59	29.9
Tertiary	77	39.1

Abbreviations: GRZ, government; LUTH, Livingstone University Teaching Hospital.

Clinical and economical characteristics of study participants are shown in Table 2 where the median (interquartile) Body Mass Index was 26.6 (23.4, 31.2), systolic blood pressure was 142.0 *mmHg* (129.0 *mmHg*, 160.0*mmHg)*, diastolic blood pressure was 88.0*mmHg* (79.0*mmHg*, 98.0*mmHg*), duration of hypertension in months was 36 (21, 120), productivity index was 0.9 (0.9, 1.0), PALYs was 45.2 (35.8, 57.0), PALY in value was $51,472.75 ($40,792.20, $64, 874.80), Cumulative PALY value was $9,804,971.00.

**Table 2:** Clinical and economic characteristics of study participants.

Characteristics	Frequency or median	Interquartile range
**Body mass index**	26.6	23.4–31.2
**SBP,** *mmHg*	142.0	129.0–160.0
**DBP,** *mmHg*	88.0	79.0–98.0
**Duration of hypertension,** *months*	36.0	21.0–120.0
**Days absent from work,** *days*, *n = 197*	2.0	0.0–14.0
**Days with presenteeism at work,** *days*	0.0	0.0–30.0
**Productivity index,** *n = 197*	0.9	0.9–1.0
**PALYs,** *n = 188*	45.2	35.8–57.0
**Cumulative PALY,** *n = 188*	8,620.9	
**PALY value** in *$*, *n = 188*	51,472.75	40,792.20–64,874.80
**Cumulative PALYs value** in *$*, *n = 188*	9,804,971.00	
**Assumed PALY without hypertension**	49.0	41.0–59.0
**Assumed cumulative PALY without hypertension**	9,387.0	
**Assumed PALY value ($) without hypertension**	55,729.66	46,630.94–67, 103.06
**Assumed cumulative PALY value ($) without hypertension**	10,676,210.58	
**PALYs lost to hypertension**	0.2	0.0–2.7
**Cumulative PALYs lost to hypertension**	766.0	
**PALYs Value ($) lost to hypertension**	342.9	0.0–3,168
**Cumulative PALYs Value ($) lost to hypertension**	871,239.58	
**Adherence**		
*Good*	103	52*
*Poor*	95	48*
**Reasons for Poor adherence,** *n = 95*		
The cost is too high	53	55.8*
I don’t work	7	7.4*
I am not aware I should take medication	4	4.2*
I am taking herbal medicines	4	4.2*
Other	27	28.4*
**Absenteeism,** *n = 197*		
*Yes*	102	51.8*
*No*	95	48.2*
**Presenteeism,** *n = 193*		
*Yes*	83	43.0*
*No*	110	57.0*

SBP, systolic blood pressure; DBP, diastolic blood pressure; PALY, productivity adjusted life years.

*percentage.

The assumed PALYs (PALYs on the same participants assuming they did not have hypertension) were 49.0 (41.0, 59.0), assumed PALYs in value was $55,729.66 (46,630.94, 67,103.06), assumed cumulative PALY in value was $10,676,210.58. The estimated PALYs lost due to hypertension was 0.2 (0.0, 2.7) bringing the cumulative PALYs lost due to hypertension to 766.0. PALYs value lost due to hypertension was 342.9(0.0, 3,168). The cumulative PALYs value lost due to the burden of hypertension was estimated to be at $871,239.58 in GDP.

Table 2. The self-reported reasons for poor adherence were the cost is too high (55.8%), participants not working (7.4%), participants unaware of the need to take medication (4.2%), on herbal medication (4.2%) and other factors (28.4%). The number of participants that reported being absent from work (percentage) was 102 (51.8%). Those that did not report being absent were 95 (48.2%), for presenteeism they were 83 (43%) and for non-presenteeism, 110 (57%).

**Fig 1:**
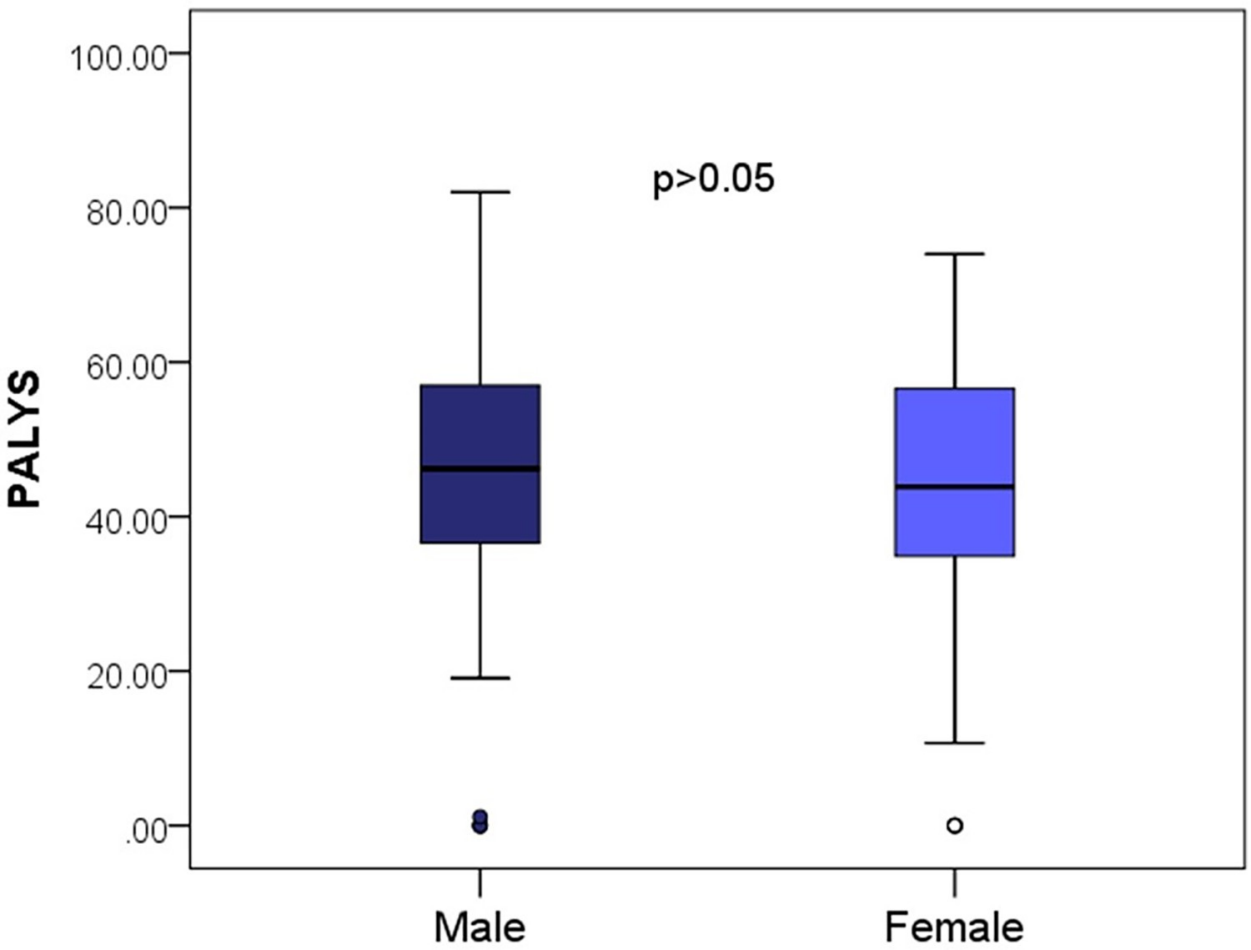
PALYs by sex. PALYs, productivity-adjusted life-years.

There was no difference in PALYs lost between males and females, Fig 1.

#### Participant characteristics associated with nonadherence to antihypertensive medication

Sociodemographic characteristics associated with nonadherence to antihypertensive medication are presented in Table 3. About 48% (n = 95; 95% confidence interval (CI) 40.8, 55.2) of the participants were nonadherent to antihypertensive medication. Participants with a younger age were more likely to be NATAM, 45 vs. 55 years, p<0.001. Of the female participants, 67.4% were nonadherent, compared to 53.4% who were adherent to antihypertensive medication (p = 0.045). A significantly higher proportion of participants from Maramba clinic (47.4%) were nonadherent to antihypertensive medication compared to those from Livingstone University Teaching Hospital (16.5%), p<0.001. A significantly higher proportion of self-employed individuals (49.5%) were nonadherent to antihypertensive medication compared to those who were nonadherent (26.2%).

**Table 3:** Sociodemographic characteristics associated with nonadherence to antihypertensive medication.

Characteristics	Adherence to antihypertensive medication		
	Yes, n (%) 103 (52)	No, n (%) 95 (48)	P value
**Age, median (IQR),** *years*	55 (45, 63)	45 (37, 56)	**<0.001**
**Sex**			
*Male*	48 (46.6)	31 (32.6)	**0.045**
*Female*	55 (53.4)	64 (67.4)	
**Facility**			
*LUTH*	86 (83.5)	50 (52.6)	**<0.001**
*Maramba*	17 (16.5)	45 (47.4)	
**Marital status, n = 196**			
*Married*	74 (73.3)	63 (66.3)	0.328
*Single*	7 (6.9)	13 (13.7)	
*Widowed*	13 (12.9)	8 (8.4)	
*Divorced*	6 (5.9)	9 (9.5)	
*Separated*	1 (1.0)	2 (2.1)	
**Employment Status**			
*GRZ employee*	24 (23.3)	15 (15.8)	**<0.001**
*Non-GRZ Employee*	10 (9.7)	12 (12.6)	
*Self employed*	27 (26.2)	47 (49.5)	
*Student*	0 (0.0)	3 (3.2)	
*Retired*	14 (13.6)	1 (1.1)	
*Unemployed (able to work)*	17 (16.5)	11 (11.6)	
*Unemployed (Unable to work)*	11 (10.7)	6 (6.3)	
**Education Status, n = 197**			
*No formal schooling*	5 (4.9)	2 (2.1)	0.124
*Primary*	25 (24.3)	29 (30.9)	
*Secondary*	26 (25.2)	33 (35.1)	
*Tertiary*	47 (45.6)	30 (31.9)	

GRZ, government; LUTH, Livingstone University Teaching Hospital; IQR, interquartile range.

Clinical and economic characteristics associated with NATAM are shown in Table 4. Factors associated with NATAM were absenteeism or days absent from work, presenteeism or days of presentism, productivity index, PALYs and PALY value.

**Table 4:** Clinical and economic characteristics associated with nonadherence to antihypertensive medication.

Characteristics	Adherence to antihypertensive medication		
	Yes, n (%) 103 (52)	No, n (%) 95 (48)	P value
**Body mass index**	27.7 (23.1, 31.2)	26.1 (23.4, 31.1)	0.332
**SBP,** *mmHg*	156 (142, 170)	141 (131, 163)	0.901
**DBP,** *mmHg*	92 (84, 101)	89 (81, 100)	0.734
**Duration of hypertension,** *months*	36.0 (12.0, 96.0)	36.0 (13.0, 102.0)	0.905
**Days absent from work,** *days*	0.0 (0.0. 12.0)	7.0 (0.0, 21.0)	**<0.001**
**Days with presenteeism at work,** *days*	0.0 (0.0, 5.5)	7.0 (0.0, 60.0)	**<0.001**
**Productivity index**	1.0 (0.9, 1.0)	0.9 (0.9, 1.0)	**<0.001**
**PALYs,** *n = 188*	52.0 (41.7, 60.5)	41.4 (33.5, 49.0)	**<0.001**
**PALY value** in *$*, *n = 188*	59,141.6 (47, 475.8, 68, 809.0)	47, 085.8 (38,196.0, 55,729.6)	**<0.001**
**Absenteeism** *n = 197*			
*Yes*	37 (35.9)	65 (69.1)	**<0.001**
*No*	66 (64.1)	29 (30.9)	
**Presenteeism,** *n = 193*			
*Yes*	28 (28.0)	55 (59.1)	**<0.001**
*No*	72 (72.0)	38 (40.9)	

SBP, systolic blood pressure; DBP, diastolic blood pressure; PALY, productivity adjusted life years.

#### Factors associated with nonadherence to antihypertensive medication in logistic regression

Table 5 shows the factors associated with NATAM at univariate and multivariable analysis. At univariate analysis, a year increase in age was significantly associated with a 6% reduced chance of being nonadherent to antihypertensive medication (odds ratio (OR) 0.94, 95%CI 0.91, 0.96). Females were 1.8 (95%CI 1.01, 3.21) times more likely to be nonadherent compared to males. Participants from Maramba Clinic were 4.55 (95%CI 2.35, 8.79) times more likely to be nonadherent to antihypertensive medication when compared to those from Livingstone University Teaching Hospital. Self-employed participants were 2.78 (95%CI 1.25, 6.19) times more likely to be nonadherent to antihypertensive medication when compared to those that are employed by the Government. Participants that reported being absent from work were 3.99 (95%CI 2.20, 7.24) times more likely to be nonadherent to antihypertensive medication when compared to those that were never absent. Participants with presenteeism at work were 3.72 (95%CI 2.04, 6.79) times more likely to be nonadherent to antihypertensive medication when compared to those that did not report presenteeism.

**Table 5:** Predictors of nonadherence to antihypertensive medications.

Variable	Odds Ratio (OR) 95% CI	P Value	Adjusted Odds Ratio (AOR) 95% CI	P value
**Age,** *years*	0.94 (0.91, 0.96)	**<0.001**	0.94 (0.90, 0.98)	**0.012**
**Sex**				
*Male*	1		1	
*Females*	1.80 (1.01, 3.21)	**0.046**	2.52 (1.18, 5.40)	**0.017**
**Facility**				
*LUTH*	1		1	
*Maramba*	4.55 (2.35, 8.79)	**<0.001**	2.01 (0.71, 5.72)	0.186
**Employment Status**				
*GRZ employee*	1		1	
*Non-GRZ Employee*	1.92 (0.66, 5.53)	0.227	3.11 (0.87, 11.05)	0.079
*Self employed*	2.78 (1.25, 6.19)	**0.012**	2.57 (1.02, 6.45)	**0.044**
*Student* *	N/A	N/A	N/A	N/A
*Retired*	0.11 (0.01, 0.96)	**0.046**	0.30 (0.03, 3.13)	0.321
*Unemployed (able to work)*	1.03 (0.38, 2.80)	0.94	1.37 (0.43, 4.35)	0.589
*Unemployed (Unable to work)*	0.87 (0.26, 2.85)	0.822	1.69 (0.38, 7.38)	0.482
**Days absent from work,** *days*	1.00 (0.99, 1.01)	0.535		
**Days with presenteeism at work,** *days*	1.01 (1.00, 1.02)	**0.009**		
**Productivity index**	0.63 (0.15, 2.64)	0.535		
**PALYs,** *n = 188*	0.95 (0.94, 0.98)	**<0.001**	1.01 (0.98, 1.05)	0.288
**PALY value** in *$*, *n = 171*	1.00 (1.00, 1.00)	**<0.001**		
**Absenteeism** *n = 180*				
*No*	1		1	
*Yes*	3.99 (2.20, 7.24)	**<0.001**	3.60 (1.16, 11.22)	**0.027**
**Presenteeism,** *n = 174*				
*No*	1		1	
*Yes*	3.72 (2.04, 6.79)	**<0.001**	0.70 (0.20, 2.40)	0.579

*had no value in one cell for the outcome.

However, at multivariable analysis only age, sex, employment status and absenteeism remained significantly associated with NATAM. A unit increase in age was significantly associated with a 6% reduced chance of being nonadherent to antihypertensive medication (OR 0.94 (95%CI 0.90, 0.98). Females were 2.52 (95%CI 1.18, 5.40) times more likely to be nonadherent compared to males. Participants that were self-employed were 2.57 (95%CI 1.02, 6.45) times more likely to be nonadherent to antihypertensive medication when compared to those that are employed by the Government. Participants that reported being absent from work were 3.60 (95%CI 1.16, 11.22, 95 CI) times more likely to be nonadherent to antihypertensive medication when compared to those that were never absent.

## Discussion

In this study we aimed to determine PALYs lost due to hypertension and determine the prevalence and correlates of NATAM. Our findings in this study show that there is a loss in PALYs amounting to 766.0 which translate to an economic loss of $871.239.58 in GDP. The proportion of participants that were nonadherent to antihypertensive medication was high and significantly associated with age, sex, facility and employment status.

Findings in our study confirm that hypertension if not controlled reduces productivity and income [2]. PALYs lost due to hypertension represent a reduction of 8.2%. These findings are similar to a study done by Hird *et al* [5], where they reported that hypertension caused a loss of PALYs. However, Hird *et al* found that the estimated PALYs of 609,801 equating to AUD$137.2 billion in lost GDP over the working lifetime were lost to hypertension [5]. Compared to our study, the study by Hird *et al* had a larger population size, as they did the research on a national level while our current study was conducted at two health facilities. Additionally, their study was futuristic as it took into account the number of PALYs that could be saved if hypertension was controlled over a lifetime in order to reduce morbidity and mortality. Ours on the other hand was a cross-sectional study and did not include futuristic information into account. A study by Tonnies *et al* in Germany had similar results despite having calculated PALYs in diabetics which usually co-exist with hypertension [37]. According to the author, depending on age and sex, PALYs lost per person with type 2 diabetes ranged between 0.3 years (men at age 69) and 12.8 years (women at age 20).

Our study also recorded a high percentage of participants who were absent from work due to hypertension at 51.8% and those that report for work but fail to work (presenteeism) were 43%. From the results above, it is evident that hypertension if left unattended would continue to reduce productivity in our economy as evidenced by several studies [2,5,38].

The prevalence of nonadherence amongst our study participants is alarming. We had an overall estimated prevalence of nonadherence to antihypertensive medication of 48%. This prevalence was lower than what was obtained in Cameroon [39] and Democratic Republic of Congo [40]. Reasons for the difference could be related to us having a smaller population size, a smaller number of participants being unemployed and a larger number of our participants having attained tertiary education. Our findings were similar to Sixty-Seven studies from 22 Asian countries in which the prevalence of non-adherence to antihypertensive medication was 48% [41]. From the information given, it is evident that nonadherence to antihypertensive medication is alarming and delays to mitigate this burden would continue to impact negatively on GDP.

In order to reduce the burden of hypertension and its economic impact, factors associated with nonadherence to medication need to be identified. From our study, age, sex, facility and employment status were associated with NATAM. A year increase in age was associated with reduced odds of being NATAM similar to one study [42] and contrary to some studies [14,43,44]. Females were more likely to be nonadherent compared to males as reported in several studies [45], however, adherence did not differ by sex in some studies [46,47]. The differences can be attributed to age, ethnicity and study designs. Other studies recruited older participants who were mostly from western/European countries and used telephone surveys and administrative databases to collect data whereas in our study, we recruited and interviewed participants in person and from sub-Sahara Africa who were mostly relatively younger. Participants that were self-employed were more likely to be nonadherent to antihypertensive medication when compared to those that are employed by the Government. Participants that reported absent for work were more likely to be nonadherent to antihypertensive medication when compared to those that were never absent. The high rate of NATAM in this current population from our study was attributed mainly (55.8%) to the high cost of medication which is an indicator of the country’s economic burden or status. In other studies, NATAM was attributed to inability to attain hypertension control, higher number of prescribed antihypertensive drugs, older age, lack of knowledge and illiteracy, having lower income, physical inactivity, cultural factors such as preference for herbal medicine as opposed to conventional medicine, and presence of comorbidities [26,48–50].

According to findings recorded by Mweene *et al* [30] in Zambia, they observed that participants were more likely to be non-adherent to antihypertensive medication if they had attained a primary level of education, had missed appointments due to lack of transport 29%, or had experienced the side effect of dizziness 28%. Patients with heart failure were more likely to be nonadherent based on the modified Hill-Bone scale, whereas those taking 3 antihypertensive drugs and those who were counseled for more than five minutes on drugs were significantly less likely to be nonadherent to antihypertensive medication. In a Korean study where more than 20% of the participants aged <45 years were more likely to be nonadherent to antihypertensive medication compared to the older ones [51]. Participants that had a lower level of education reported a higher proportion of nonadherence (16.8%) when compared to those who had a higher level of education and participants with less engagement in economic activities were more likely to be nonadherent to antihypertensive medication [51].

### Future perspectives and public health implications

From our study some questions remain unanswered and we recommend future studies to consider them. For example, the actual specific adverse outcomes resulting from NATAM that have an impact on PALYs must be considered to aid policy makers and researchers in coming up with better interventions. Medication adherence level or rate and the types of adherence measures are crucial in providing comprehensive information about the nature and magnitude of NATAM. The majority of studies from literature use cross-sectional designs. More prospective studies are required to better understand the incidence of NATAM and its effect on PALYs.

Hypertension is a known risk factor for cardiovascular diseases, end-organ damage, disability and death. NATAM accelerates the incidence of these adverse outcomes. The diminished quality of life and loss of PALYs due to morbidity has an economic and public health implication. The more PALYs lost due to hypertension the more GDP per effective worker is lost. This results in an unstable or poor economy incurring high cost of medication, inability to increase and improve health infrastructure and service delivery, more demand for primary health care due to increased adverse outcomes associated with NATAM leading to a higher burden of morbidity and mortality.

### Strengths and limitations of the study

PALYs provide economic evaluations and complementary methods of estimating the potential benefit of health interventions. We used GDP per effective worker to reflect the economic cost of each PALY as done in other studies. However, we did not account for the various economic strata before directly multiplying with the per capita GDP of Zambia to obtain the results and economic losses. Therefore, our findings are not generalizable to Zambia or the Livingstone district as they are only indicative of estimated losses associated with productivity likely due to hypertension in a small population studied.

A range of variables were included to give the study a wider scope and understanding of the factors surrounding NATAM. This study highlights how much GDP is lost due to hypertension and the need to quickly curb the condition

The population of persons living with hypertension who routinely attend hypertension clinics is small. Therefore, the results may not be generalized beyond the study sites. Although this study determined socioeconomic factors associated with NATAM, being a cross-sectional study, we were not able to determine causality.

## Conclusions

Our study highlights a high economic loss of PALYs due to hypertension suggesting a significant negative impact on GDP per effective worker in a population of adult Zambians from Livingstone. The estimated PALYs lost due to hypertension in our study is a reflection of one of the fundamental and key aspects of uncontrolled blood pressure due to NATAM. We found a significant proportion of persons living with hypertension were non-adherent to antihypertensive medication and hence at risk for adverse outcomes. NATAM is a predictor of morbidity and mortality in persons with hypertension and therefore an index that potentially impacts PALYs negatively as it affects productivity among individuals of working age. Therefore, there is need for clinical and public health interventions that target these aspects in the overall agenda to mitigate the burden and ameliorate the health-economic adverse effects of hypertension.

## Supporting information

S1 Table Strobe checklist. (DOCX)

S1 File Dataset. Hypertension PALYs DATA.
(XLSX)
